# Comparative Genomic and Regulatory Analyses of Natamycin Production of *Streptomyces lydicus* A02

**DOI:** 10.1038/s41598-017-09532-3

**Published:** 2017-08-22

**Authors:** Huiling Wu, Weicheng Liu, Lingling Shi, Kaiwei Si, Ting Liu, Dan Dong, Taotao Zhang, Juan Zhao, Dewen Liu, Zhaofeng Tian, Yuesen Yue, Hong Zhang, Bai Xuelian, Yong Liang

**Affiliations:** 1 Institute of Plant and Environment Protection, Beijing Academy of Agriculture and Forestry Sciences, Beijing, 100097 China; 2 Beijing Research and Development Center for Grass and Environment, Beijing Academy of Agriculture and Forestry Sciences, Beijing, 100097 China; 3 BGI-Shenzhen, Shenzhen, Guangdong, 518083 China

## Abstract

*Streptomyces lydicus* A02 is used by industry because it has a higher natamycin-producing capacity than the reference strain *S*. *natalensis* ATCC 27448. We sequenced the complete genome of A02 using next-generation sequencing platforms, and to achieve better sequence coverage and genome assembly, we utilized single-molecule real-time (SMRT) sequencing. The assembled genome comprises a 9,307,519-bp linear chromosome with a GC content of 70.67%, and contained 8,888 predicted genes. Comparative genomics and natamycin biosynthetic gene cluster (BGC) analysis showed that BGC are highly conserved among evolutionarily diverse strains, and they also shared closer genome evolution compared with other *Streptomyces* species. Forty gene clusters were predicted to involve in the secondary metabolism of A02, and it was richly displayed in two-component signal transduction systems (TCS) in the genome, indicating a complex regulatory systems and high diversity of metabolites. Disruption of the *phoP* gene of the *phoR-phoP* TCS and *nsdA* gene confirmed phosphate sensitivity and global negative regulation of natamycin production. The genome sequence and analyses presented in this study provide an important molecular basis for research on natamycin production in *Streptomyces*, which could facilitate rational genome modification to improve the industrial use of A02.

## Introduction

*Streptomyces lydicus* A02 is a Gram-positive filamentous actinomycete that produces natamycin (also known as pimaricin), which is the only member of the polyene antibiotic family that does not exert its antifungal action by forming pores and permeabilizing the plasma membrane^1, 2^. It inhibits the growth of fungi via the immediate inhibition of amino acid and glucose transport across the plasma membrane. Due to its broad spectrum of antifungal activity and naturally occurring fungal resistance to natamycin being exceptionally rare, natamycin is widely used in the treatment of fungal keratitis, as a food preservative and as an antifungal agent in agriculture^1, 3, 4^. In addition to *S*. *lydicus*, three other species of *Streptomyces* are known to produce natamycin, i.e., *S*. *natalensis*, *S*. *gilvosporeus*, and *S*. *chattanoogensis* ^3, 5, 6^. Despite extensive studies on their natamycin biosynthetic pathways, the underlying mechanisms of natamycin production and the regulatory role at the genomic level in these *Streptomyces* species remain unclear.

The natamycin biosynthetic gene cluster (BGC) was first characterized in *S*. *natalensis* ATCC 27448^3^. It contains 5 giant multi-enzyme proteins (PimS0-PimS4) and 11 enzymes involved in polyene tailoring or export (PimA-PimK). Additionally, a series of regulators (PimR, PimM, and PimT) are associated with the biosynthesis of natamycin^7–10^. In 2011, the natamycin BGC of *S*. *chattanoogensis* L10 was characterized^11^. A comparative cluster analysis of the two strains revealed the presence of different architectures and indicated the occurrence of the evolution of antibiotic BGCs.

Several studies have reported the role of various regulators in natamycin biosynthesis, such as the pathway-specific regulators PimM (ScnRII in *S*. *chattanoogensis* and SlnM in *S*. *lydicus* A02)^1, 9, 12^, PimR^8^, and PimT^10^; the phosphopantetheinyl transferases^13^; the pleiotropic regulator SngA^14^; the *phoR-phoP* two-component system (TCS)^15^; the pleiotropic regulator AdpAch^12^, and the γ-butyrolactone autoregulator receptor SngR^16^. Cloning of new regulatory genes and exploration of novel regulatory mechanisms are still ongoing; however, the complete genome sequence of a natamycin-producing strain has not been reported to date.

The wild-type strain A02 displayed obvious differences in morphology, pigments and natamycin production compared to *S*. *natalensis* ATCC 27448 and *S*. *chattanoogensis* NRRL B-2255. A higher natamycin-producing capacity than that of *S*. *natalensis* ATCC 27448 and *S*. *chattanoogensis* NRRL B-2255 was detected in A02 (Supplementary Fig. S1). We have constructed a series of engineered strains, including overexpression of the *slnM* gene with different promoters (ermEp* promoter, native promoter and dual promoters), heterologous expression of *Vitreoscilla* haemoglobin and coexpression of *Vitreoscilla* haemoglobin and *Bacillus megaterium* glucanase, to improve the natamycin yield or antifungal metabolites in strain A02^1, 17^.

In this study, we completed the whole-genome sequencing of *S*. *lydicus* A02 and extended these studies to the genomes of eight other *Streptomyces* species – *S*. *venezuelae* ATCC 10712, *S*. *bingchenggensis* BCW-1, *S*. *auratus* AGR0001, *S*. *avermitilis* MA-4680, and *S*. *coelicolor* A3(2) – each of which has more than 400 proteins matched to the A02 genome according to the gene annotation. *Streptomyces lydicus* 103 has not been reported to be able to produce natamycin, but it is the only corresponding species genome available from public databases^18^. The natamycin-producing strains are *S*. *natalensis* ATCC 27448 and *S*. *chattanoogensis* NRRL ISP5002, both genome sequences of which were recently produced by whole-genome shotgun sequencing.

The phylogenetic trees were constructed based on 16 S rRNA, the core-pan genome and gene families. We identified the gene synteny across the whole genome between A02 and the other eight *Streptomyces* species and provide a comparative analysis of natamycin BGC in three natamycin-producing strains. The role of *phoR*-*phoP* TCS and the global negative regulatory gene *nsdA* in natamycin production was also evaluated. The genome sequence and analyses presented in this study provide important evidence of the molecular basis for further research on natamycin and other valuable secondary metabolites produced by strain A02.

## Results and Discussion

### Genome sequencing and assembly of *S*. *lydicus* A02

Initially, a total of 2,507 Mb of raw data were produced from the HiSeq. 2000 sequencing platform, from 43,361 reads. The assembly consisted of 1 contig of 9,300,345 bp (Supplementary Table S1). After single-molecule real-time sequencing (SMRT) and data clean-up, 593.1 Mb of sequence data were obtained, with an average sub-read length of 7.3 kb (Supplementary Table S2). During preassembly, 4 kb was chosen as the cutoff for recruiting seed reads, and 383 Mb of seed reads were generated. After read correction, a 263 Mb preassembly with an average read length of 6.6 kb was obtained (Supplementary Fig. S2). The final assembly was composed of a chromosomal scaffold of 9,307,519 bp (70.67% G + C) (Table 1, Supplementary Fig. S3) without any plasmid.

**Table 1:** Characteristics of the *S*. *lydicus* A02 genome assembly using single-molecule real-time sequencing.

Genome Size (bp)	9,307,519
GC Content (%)	70.67
Gene Number (#)	8,888
Total Length (bp)	8,035,014
Gene Average Length (bp)	904
Gene Length/Genome (%)	86.33
GC Content in Gene Region (%)	68.15
Intergenic Region Length (bp)	1,272,505
GC Content in Intergenic Region (%)	68.15
Intergenic Region Length/Genome (%)	13.67
Tandem Repeat Number	2,217
Total Length (bp)	99,927
Range of Repeat Size (bp)	4-828
Tandem Repeat Length/Genome (%)	1.0736
Minisatellite DNA Number	1,451
Microsatellite DNA Number	247
rRNA Number	18
tRNA Number	68
sRNA Number	14

### Genome annotation and general features of *S*. *lydicus* A02

There are 8,888 protein CDSs accounting for 86.33% of the complete genome. A total of 3,921 putative proteins were matched to the genome sequence of *S*. *auratus* AGR0001, which produces neophoslactomycin A and possesses potent biocontrol activity against certain phytopathogenic fungi^19^. Additionally, 864 hypothetical proteins have no match to any known protein databases. More than 400 putative proteins were matched to five different *Streptomyces* species, *S*. *venezuelae* ATCC 10712, *S*. *bingchenggensis* BCW-1, *S*. *auratus* AGR0001, *S*. *avermitilis* MA-4680, and *S*. *coelicolor* A3(2). A total of 68 tRNA-encoding genes and six rRNA operons (six 5 S rRNAs, six 23 S rRNAs and six 16 S rRNAs) were found in the linear chromosome.

A comparative analysis was performed on the basic genome features of A02 and 11 previously sequenced *Streptomyces* strains (Table 2). The genome of *S*. *lydicus* A02 is smaller than the previously sequenced linear genome of *S*. *bingchenggensis* BCW-1 (11.9 Mbp) and *S*. *hygroscopicus* 5008 (10.15 Mbp) but larger than those of other *Streptomyces* strains^19–23^. However, it has a gene number that is lower than that of only *S*. *bingchenggensis* BCW-1. The genome sequence of *S*. *lydicus* A02 has a G + C content that is comparable to that of *S*. *avermitilis*, which is higher than *S*. *pristinaespiralis* 25486 and *S*. *natalensis* ATCC 27448 but lower than the other strains. Among all these strains, *S*. *lydicus* A02 has the smallest average CDS size (904 bp).

**Table 2:** Comparison of key genomic features among the twelve fully assembled genomes of *Streptomyces*.

Species	Genome (Mp)	GC content	Gene number	Average CDS (bp)	Plasmids	tRNA	rRNA operon
*S*. *lydicus* A02	9.3	70.67%	8,888	904	0	68	6
*S*. *auratus* AGR0001	7.85	71.5%	7,102	NA	0	66	8
*S*. *bingchenggensis* BCW-1	11.94	70.8%	10,107	1,031	0	66	6
*S*. *natalensis* ATCC 27448	8.65	70.6%	7,564	NA	0	70	3
*S*. *avermitilis* MA-4680	9.03	70.7%	7,582	1,027	1	68	6
*S*. *hygroscopicus* 5008	10.15	71.9%	8,849	952	2	68	6
*S*. *coelicolor* A3	8.67	72.1%	7,825	991	2	63	6
*S*. *cattleya* 8057	8.09	72.99%	7,580	NA	1	64	6
*S*. *venezuelae* 10712	8.23	72.4%	7,536	NA	0	67	7
*S*. *pristinaespiralis* 25486	8.13	69.7%	7,025	NA	0	65	7
*S*. *chattanoogensis* NRRL ISP-5002	9.13	70.9%	8031	NA	0	67	10
*S*. *lydicus* 103	8.2	NA	7128	NA	0	68	7

The distribution of genes in *S*. *lydicus* A02 into COG functional categories is presented in Supplementary Table S3. CRISPRs compose a type of immune system that is widespread in bacteria and archaea to protect cells against foreign genetic elements (e.g., plasmids and phages)^24^. Three credible and several questionable CRISPR loci were detected in the chromosome of strain *S*. *lydicus* A02. The components of the CRISPR elements were identified, and we conducted comparative analysis of the repeats and spacers with those identified in the GenBank database, prediction of the leader region in the CRISPR locus and Cas genes and related analyses (Supplementary Fig. S4).

### Phylogenetic analyses of streptomycetes

**Figure 1:**
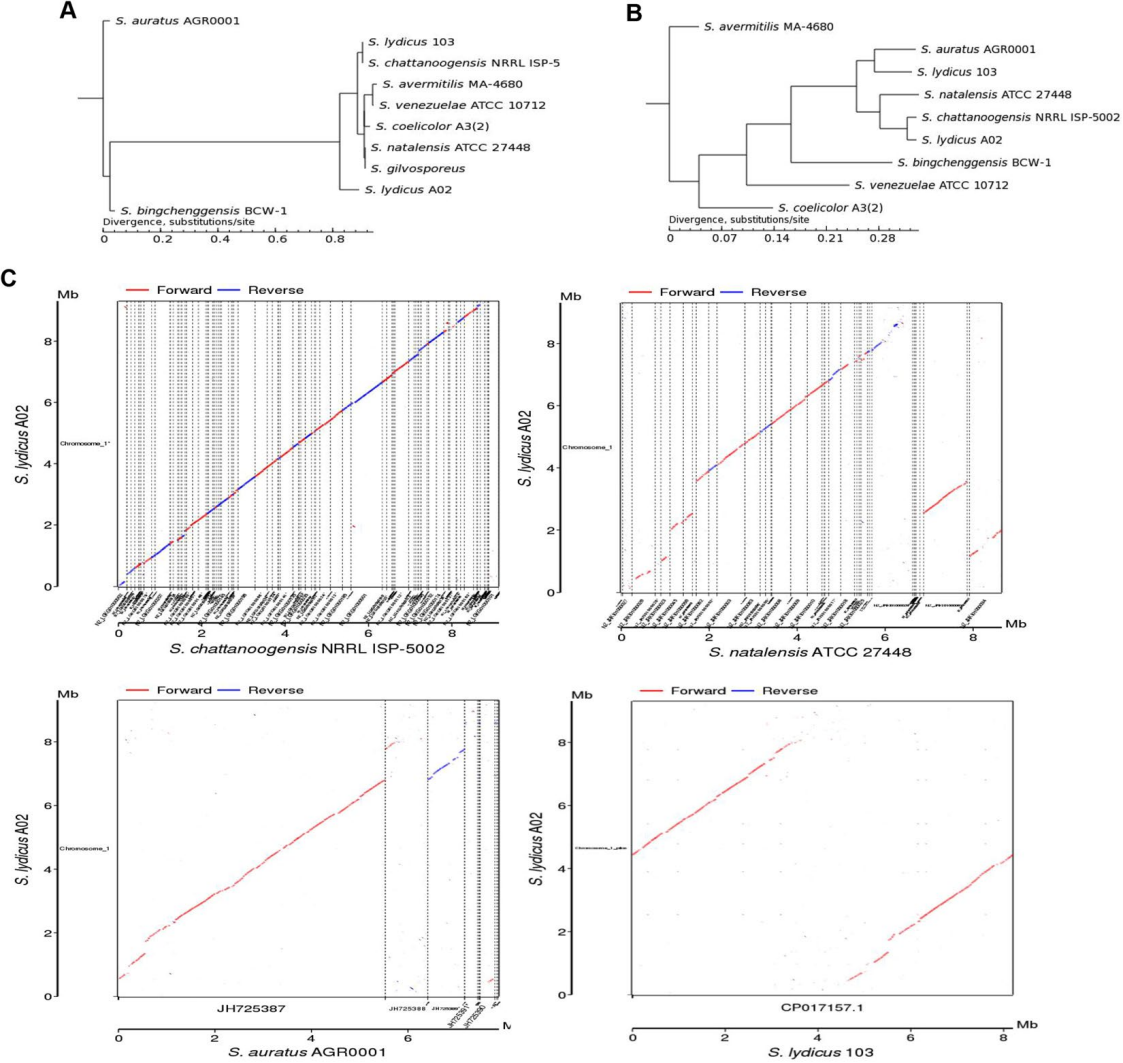
Phylogenetic trees for streptomycete. The phylogenetic tree is constructed by TreeBeST using PHYML based on (**A**) the 16 S rRNA gene and (**B**) the phylogenetic tree analysis’s result based on SNP analysis (core-fan genome and gene family).

To infer the phylogenetic relations of A02 and other *Streptomyces* strains, we constructed phylogenetic trees based on 16 S rRNA, gene family and core-pan genome. In the phylogenetic tree based on the 16 S rRNA gene sequences (Fig. 1A), 10 strains were primarily grouped into two subgroups. Strain A02 was positioned into a single clade that is equally closely related to *S*. *lydicus* 103 and *S*. *chattanoogensis* NRRL ISP5002, which were tightly grouped. Two other natamycin producing strains, *S*. *natalensis* ATCC 27448 and *S*. *gilvosporeus* ATCC 13326, were classified into the same group. However, the internal branches of the tree that were based on the 16 S rRNA gene sequences had low bootstrap support^25^. Single-gene phylogenies might not always reflect the evolutionary history of a species due to the high degree of lateral gene transfer^26^. We also reconstructed the phylogenetic trees using the core-pan genome and gene families (Fig. 1B). Both the trees exhibited similar identities, with larger bootstrap scores and higher robustness than that of 16 S rRNA. *S*. *gilvosporeus* was not included in this tree because its genome sequence is not available in any public database. The tree of the core-pan genome and gene families suggested that all the *Streptomyces* strains were clustered within one clade except *S*. *avermitilis* MA-4680. Strains A02 and *S*. *chattanoogensis* NRRL ISP5002 are tightly grouped, and they are equally closely related to *S*. *natalensis* ATCC 27448. *S*. *auratus* AGR0001 and *S*. *lydicus* 103 were classified into the same group. The MUMmer-based whole-genome alignment revealed that the sequence of strain A02 aligned at a relatively high level with *S*. *chattanoogensis* NRRL ISP5002, showing minimal genomic rearrangements when compared with other *Streptomyces* species (Fig. 1C). The average nucleotide identity (ANI) for 9 *Streptomyces* genome sequences was calculated using the BLAST algorithm and MUMmer alignment using the software tool JSpecies^27^. The ANI value between A02 and *S*. *chattanoogensis* NRRL ISP5002 was 97.4% (Supplementary Table S4), indicating that the two strains share the closest phylogenetic relations. The ANI value between A02 and other *Streptomyces* suggested similar phylogenetic relations between A02 and other *Streptomyces* with the gene families and core-pan genome. The phylogenetic analyses of streptomycetes based on the core-pan genome, gene families and ANI value showed that *Streptomyces* species that can produce natamycin are more frequently associated with genetic recombination, which may reduce the divergence of these bacteria.

### Core and pan-genome analyses

**Figure 2:**
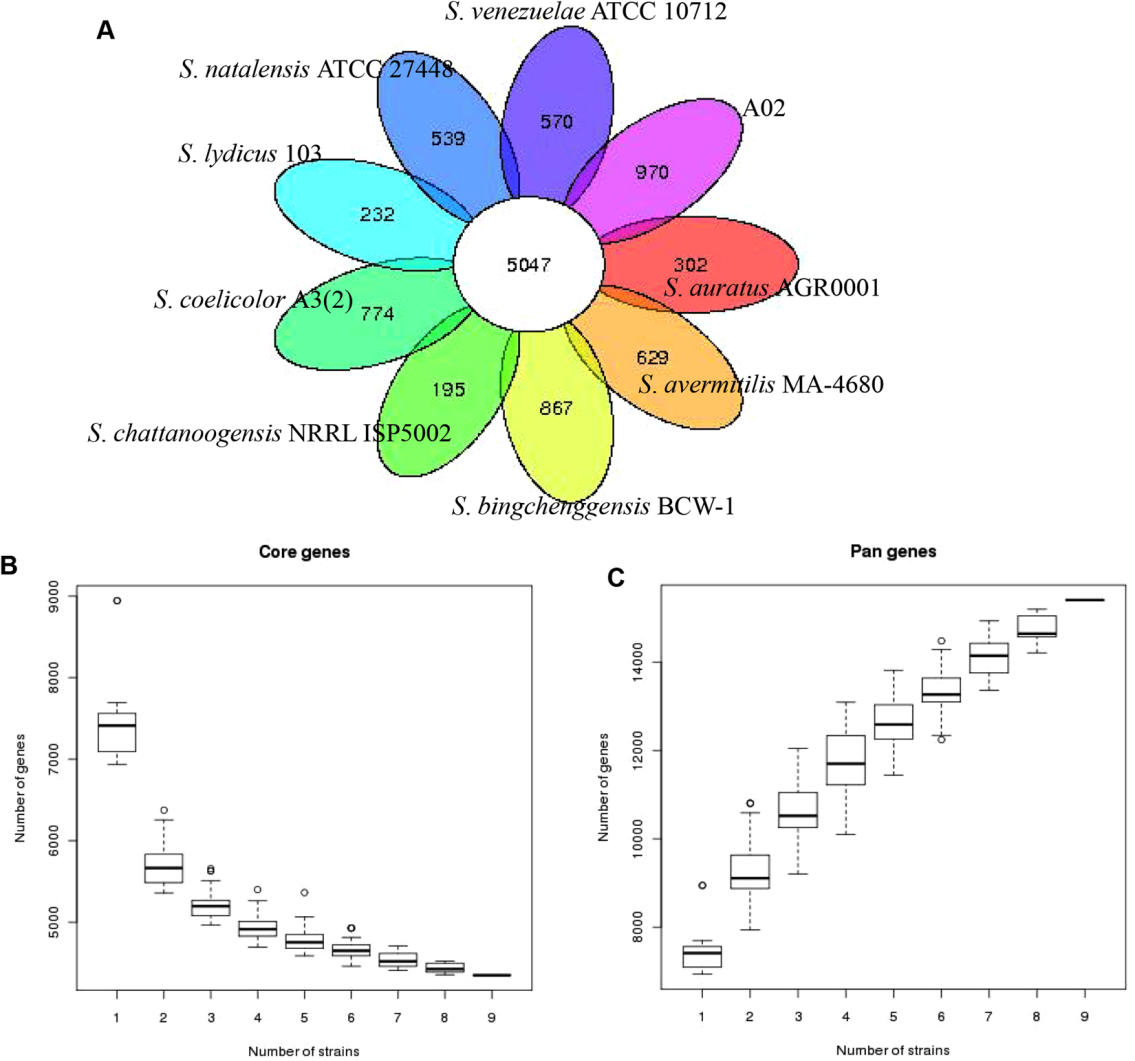
The pan-genome of streptomycetes. (**A**) Flower plots showing the core gene number and strain-specific gene number in 9 streptomycete strains. (**B**) Dilution curve of 9 streptomycete pan and core genomes.

The pan-genome defines the entire genomic repertoire of a given phylogenetic clade and encodes for all possible lifestyles of its organisms, including the core genome, dispensable genome and strain-specific genes^28^. To understand the genetic composition of the 9 streptomycetes strains in the core-pan genome more thoroughly, we clustered all 69,446 protein CDSs in 9 streptomycetes strains. All the protein CDSs were clustered into 15,404 orthologues; 5,047 (32.8%) orthologues were identified in 9 strains as the streptomycetes core genome (Fig. 2A). The 5,279 orthologues were identified as dispensable genomes, and 5,087 genes were strain-specific. *S*. *chattanoogensis* NRRL ISP5002 had the smallest number of strain-specific genes because it has the closest phylogenetic relationship with A02 based on the gene family, core-pan genome and whole-genome alignment. *S*. *lydicus* 103 had a smaller number of strain-specific genes because of the phylogenetic relationship between *S*. *lydicus* 103 and A02. *S*. *auratus* AGR0001, as the strain with the smallest genome size in these *Streptomyces* species, matched the largest number of genes to the *S*. *lydicus* A02 genome but also displayed a smaller number of strain-specific genes. However, *S*. *bingchenggensis* BCW-1 had the largest number of strain-specific genes, perhaps because it has the largest genome size and gene numbers, although it does not have the greatest difference from the *S*. *lydicus* A02 genome.

To understand the relationship between the streptomycetes pan-genome size, the core genome number and the strain number, we plotted the pan-genome profile fitted curves of the nine *Streptomyces* strains. As shown in Fig. 2B, we could intuitively observe that the more genomes we added, the more new orthologue clusters were discovered, implying an open pan-genome of these *Streptomyces* strains. The number of core genomes would be expected to converge to a constant value, as judged from the slope of exponential decay (Fig. 2C). A heat map illustrated the distribution of the genes after core gene deletion (Supplementary Fig. S5).

### Comparative cluster analysis of natamycin BGCs

**Figure 3:**
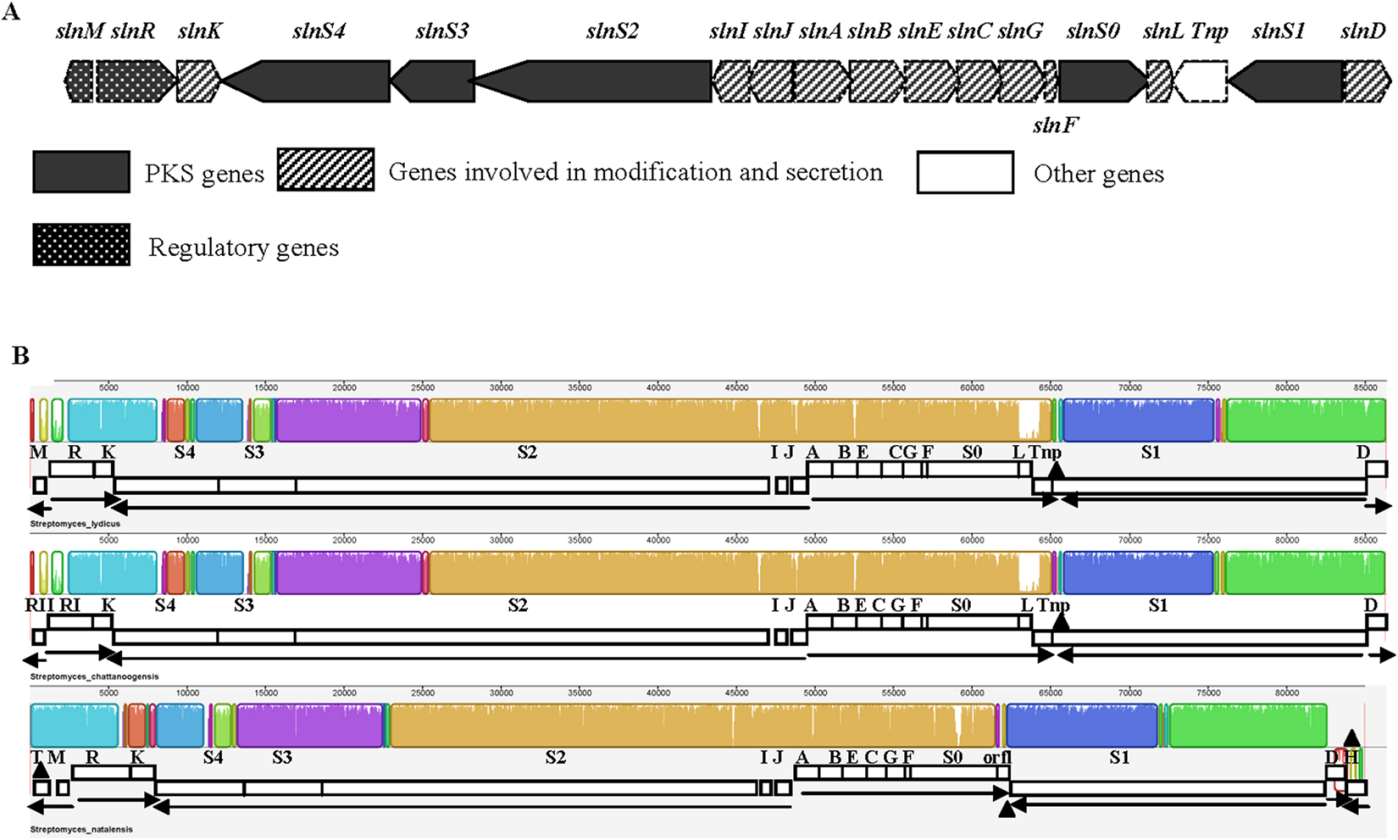
The natamycin biosynthetic gene cluster (BGC) of A02 (**A**) and comparison analysis of the natamycin BGCs of sln, scn and pim using the MAUVE program (**B**). The horizontal panels depict the sln (upper), scn (middle) and pim (lower) clusters. Mean sequence similarities are proportional to the heights of the red bars, and the respective scales show the sequence coordinates in base pairs. Regions with low similarities and strain-specific regions are marked with black triangles. Arrows indicate deduced transcriptional units.

The natamycin BGC of *S*. *lydicus* A02 contains 19 open reading frames (ORFs) putatively involved in natamycin biosynthesis. It contains all the ORFs reported in *S*. *chattanoogensis* L10, including 5 polyketide synthase genes (*slnS0*, *slnS1*, *slnS2*, *slnS3*, and *slnS4*), 11 post-tailoring genes (*slnA*, s*lnB*, *slnC, slnD*, *slnE*, *slnF*, *slnG, slnH*, *slnI*, *slnJ* and *slnK*), and 2 regulator genes (*slnM* and *slnR*) (Fig. 3A).

The product of the *slnM* has 100% identity to ScnRII and 96% identity to PimM, which are predicted to encode putative RNA polymerase sigma factor in *S*. *chattanoogensis* L10 and *S*. *natalensis* ATCC 27448. The predicted gene *slnD* encodes a putative methyltransferase, which has 99% identity to ScnD in *S*. *chattanoogensis* L10 and 97% identity to PimD in *S*. *natalensis* ATCC 27448. The gene *scnRII* is the left boundary, and *scnD* is the right boundary of the natamycin BGC according to Du *et al*.^12^. The deduced functions of the natamycin BGC genes and the results of similarity comparative analysis are listed in Supplementary Table S5. To facilitate a comparative analysis with the natamycin BGCs of *S*. *natalensis* (pim cluster) and *S*. *chattanoogensis* (scn cluster), we named the cluster from A02 the sln cluster.

The complete sequences of the three natamycin BGCs were aligned in the MAUVE program^29^. Based on BlastP searches, the natamycin BGC of *S*. *lydicus* A02 has an organization that is similar to that of *S*. *chattanoogensis* L10 as described by Du *et al*.^12^. Orthologous genes from the three natamycin BGCs are located in identical relative positions, and genes from the sln and scn clusters show a higher sequence identity than do those from the sln and pim clusters at the protein level. In contrast to the sln cluster, the pim cluster contains 2 major strain-specific regions at the two ends (Fig. 3B), i.e., pimH, putatively involved in natamycin export^7^, and pimT, involved in modulating the expression of natamycin biosynthetic genes via secretion of the natamycin inducer factor^10^. The scn cluster has no major strain-specific region compared with the sln cluster.

Similar to the scn cluster, the sln cluster contains a putative transposase gene (*tnp*) located between *slnL* and *slnS1*, which is likely involved in horizontal gene transfer of the natamycin BGC. Except for the putative transposase (97%), all the remaining putative proteins of the natamycin BGCs in *S*. *lydicus* A02 show significantly high similarity (≥99%) to their counterparts in *S*. *chattanoogensis* L10, suggesting that the two strains may share the same metabolic regulatory mechanisms in the natamycin biosynthetic pathway.

### Secondary metabolism

Forty secondary metabolite biosynthetic gene clusters were predicted in the complete genome of A02, including thirteen polyketide (PKS) and non-ribosomal polyketide (NRPS) clusters, six terpenoids, five lantipeptides, three siderophores, two butyrolactones, two lassopeptides, one cyanobactin, bacteriocin, ectoine, ladderane, melanin, and oligosaccharide, and three other undefined clusters (Supplementary Table S6). The total length of these gene clusters occupied 15.6% of the genome, specifically with total lengths of 1151.7 kb in the arm region and 299 kb in the core region. In contrast to the essential genes, most of the secondary metabolite biosynthetic gene clusters (30 out of 40) were in the non-core regions. The largest predicted gene cluster was for natamycin biosynthesis and was located in the 10.7 Mb right-arm region of the chromosome.

Among the thirteen PKS and NRPS clusters, most of the CDSs for PKSs and NRPSs of ten clusters have orthologues in the *S*. *chattanoogensis* L10, with the best hitting scores by comparing against the NCBI database via BLASTP (Supplementary Table S6). The other three clusters showed no orthologues in the *S*. *chattanoogensis* genome, i.e., T2PKS (the first cluster) and NRPS (the sixteenth cluster) in the non-core regions and T1PKS (the twenty-ninth cluster) in the core region. Most cluster-spanning areas showed high nucleotide sequence identity to *S*. *lydicus* 103, except for the natamycin biosynthetic gene cluster (the thirty-fifth) and chattamycin biosynthetic gene cluster (the thirty-seventh). An analysis of the secondary metabolic gene clusters indicated a high diversity of metabolites in A02 and complex phylogenetic relationships between A02, *S*. *lydicus* 103 and *S*. *chattanoogensis* L10.

### Two-component signal transduction system (TCS)

Bacterial TCSs consist of a sensor histidine kinase (HK) and a response regulator/transcription factor (RR). TCSs play important roles in detecting and responding to diverse environmental stresses and cellular changes^30^. The genome of *S*. *lydicus* A02 contains a large number of TCSs. Based on the BlastP analysis and conserved domains of known HKs and RRs, more than 100 *hk* and *rr* genes were discovered throughout the entire chromosome. Based on the homology-box, the topological feature of HK and the architecture of the C-terminal output domain of RR^31^, 61 TCS pairs (Supplementary Table S7) of A02 were grouped into 4 previously described subfamilies. There are 31 HK/RR pairs of the NarL subfamily, 26 pairs of the OmpR subfamily, 3 pairs of the CitB subfamily, and 1 pair of the AmtB subfamily. The complex and extensive regulatory systems indicate the high diversity of metabolites in *S*. *lydicus* A02.

It has been reported that phosphate control of actinorhodin and undecylprodigiosin biosynthesis in *S*. *lividans* is mediated by the two-component *phoR*-*phoP* system^32^. Additionally, phosphate decreases the production of pimaricin and negatively regulates the expression of the pimaricin biosynthesis genes in *S*. *natalensis*. Disruption of *phoP* resulted in increased pimaricin production and reduced sensitivity to phosphate control in *S*. *natalensis* ^15^. Sequence analysis showed that the *phoR*-*phoP* TCS also exists in *S*. *lydicus* A02 (encoded by A02_3630/3632). They have a similar genetic organization and share high similarity both at the nucleotide and the amino acid levels with the *phoR*-*phoP* cluster found in other *Streptomyces* strains. The genes *phoR* (1,272 bp) and *phoP* (672 bp), which encode proteins of 424-aa and 224-aa, respectively, share 94/96% identity in nucleotide sequence and 97/99% similarity at the amino acid level with those in *S*. *natalensis* (Supplementary Table S8).

**Figure 4:**
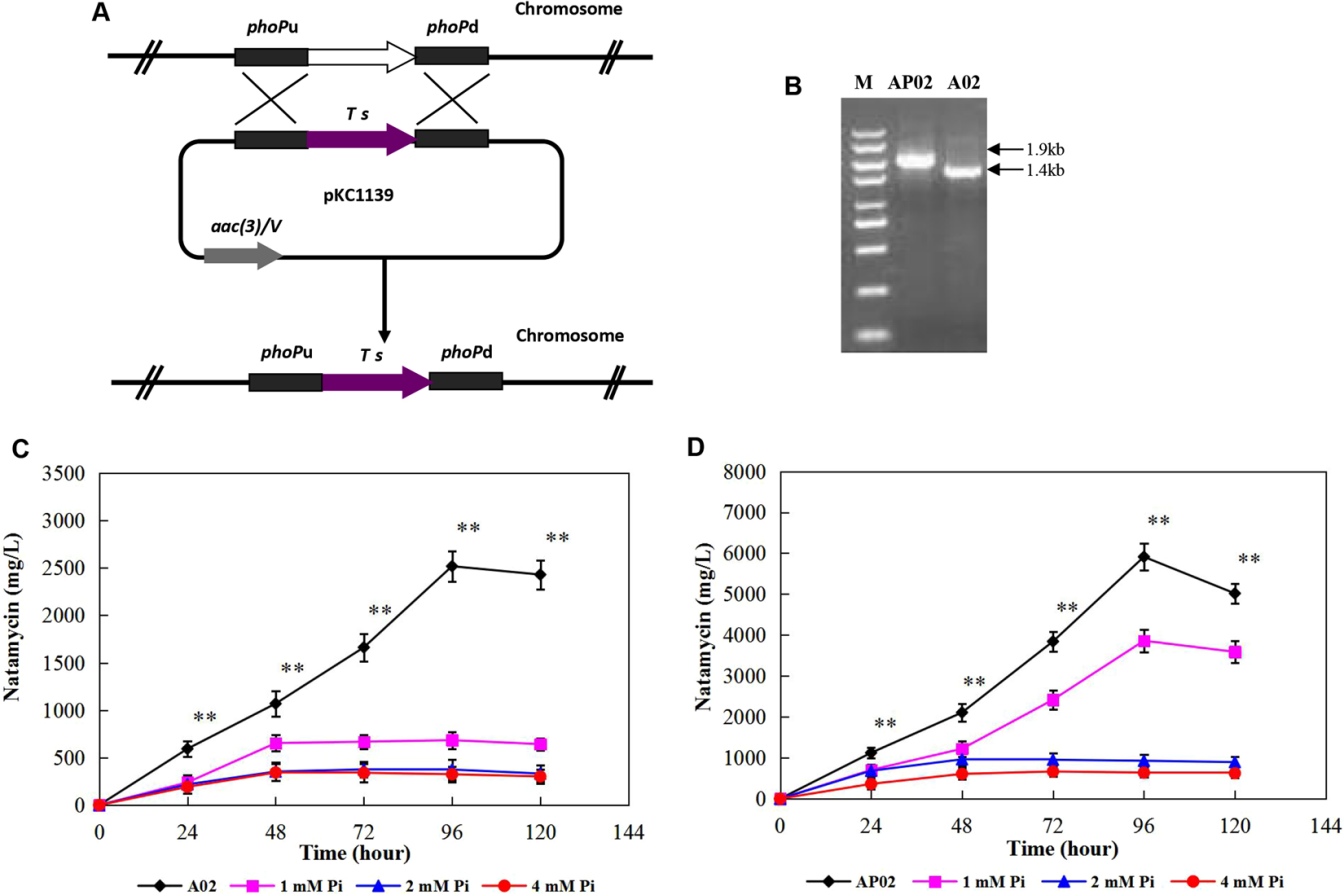
Construction of the *phoP* mutant and the effect of phosphate concentration on natamycin production in A02. (**A**) Schematic showing the deletion of *phoP* in A02. (**B**) PCR verification of *phoP* mutant. Lane M, DNA Marker. (**C**) Effect of increasing phosphate concentrations on natamycin production in A02. (**D**) Effect of increasing phosphate concentrations on natamycin production in AP02. The experiments were repeated three times, and the data shown are the mean ± SE; *P < 0.05, **P < 0.01.

To verify the function of *phoP* in natamycin biosynthesis in *S*. *lydicus* A02, the *phoR*-*phoP* system was cloned according to the annotated sequence from *S*. *lydicus* A02, and a *phoP*-disrupted mutant was obtained. A 535-bp fragment internal to the chromosomal *phoP* gene was replaced with a thiostrepton resistance (*tsr*) gene, generating an apramycin-sensitive, thiostrepton-resistant mutant (named AP02) (Fig. 4A). Exconjugants with double crossovers were confirmed by PCR using PP1 and PP2 as primers and genomic DNA from A02 and AP02 as templates. The expected 672-bp *phoP* fragment was amplified from strain A02, while the PCR product from the double-crossover recombination mutant AP02 was 1.2-kb larger than that from the wild-type A02 (Fig. 4B). These results showed that *phoP* was precisely replaced with the *tsr* gene in the homologous double-crossover recombination mutant AP02. The production of natamycin in the *phoP*-disrupted mutant was approximately 2.3-fold of the wild-type A02 in YSG medium, and it also showed reduced sensitivity to phosphate control (Fig. 4C,D). At 1 mM phosphate, the *phoP* mutant still produced approximately 65% of the natamycin level in the unsupplemented cultures in YSG medium, whereas the wild-type A02 was unable to synthesize significant amounts of natamycin at this phosphate concentration. This result indicated that, similar to *S*. *natalensis*, the phosphate control of natamycin biosynthesis in *S*. *lydicus* A02 was mediated by the two-component *phoR*-*phoP* system, which plays a negative regulatory role in natamycin biosynthesis.

### *NsdA* in *S*. *lydicus* A02 and its regulatory role in natamycin biosynthesis

The *nsdA* gene, which was first found in *S*. *coelicolor* A3(2) in 2006, is widely present and conserved in *Streptomyces* and negatively influences antibiotic production in multiple species of actinomycetes^33–35^. The disruption of *nsdA* in *S*. *coelicolor* resulted in an overproduction of spores and three antibiotics, including actinorhodin, methylenomycin, and calcium-dependent antibiotic. An *nsdA*-disrupted mutant of *S*. *bingchengensis* displayed increased production of milbemycin A4 (1.5-fold) and nanchangmycin (9-fold), with more pigment and spores than its wild-type strain.

The genome sequence of *S*. *lydicus* A02 encodes a predicted homologue of *nsdA* (with 88% identity to *nsdA* in *S*. *qingfengmyceticus*) that occurs in regions having a high degree of synteny with the respective chromosomal locations in *S*. *qingfengmyceticus*. Based on its predicted sequences, the *nsdA* in *S*. *lydicus* A02 was amplified using the primers NP1 and NP2. The amplified 1.479-kb *nsdA* gene encodes a 493-amino-acid protein, which shares 80-88% identity in nucleotide sequence and 75-91% similarity at the amino acid level with *nsdA* in the other nine *Streptomyces* strains (Supplementary Table S9).

**Figure 5:**
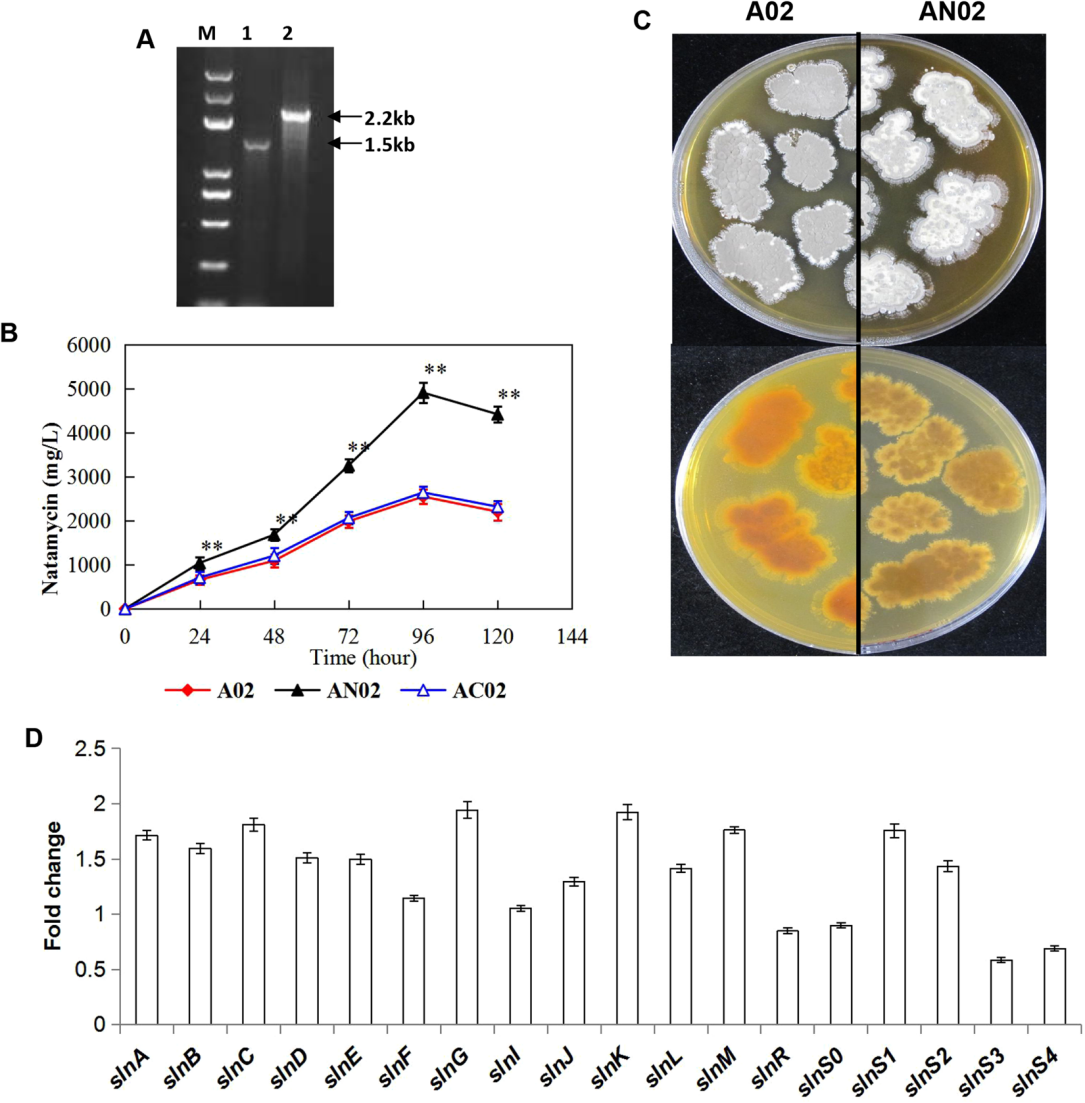
Effects of *nsdA* disruption on natamycin production and expression of the sln cluster in A02. (**A**) PCR verification of the *nsdA* mutant. Lane M, DNA Marker; Lane 1, Amplification with primers NP1 and NP2 in A02; Lane 2, Amplification with primers NP1 and NP2 in AN02. (**B**) Spore formation (upper) and pigment production (lower) of the A02 and AN02 on PDA plates but different sides for 10 days. (**C**) Natamycin production in the A02, AN02, and AC02. The experiments were repeated three times, and the data shown are the mean ± SE; *P < 0.05, **P < 0.01. (**D**) Expression of the genes of the sln cluster detected by qRT-PCR in A02 and AN02.

To study the role of *nsdA* in natamycin biosynthesis in *S*. *lydicus* A02, a 972-bp fragment internal to the chromosomal *nsdA* gene was replaced with a *tsr* gene, generating an apramycin-sensitive, thiostrepton-resistant mutant (AN02). Exconjugants with double crossovers were confirmed by PCR, using NP1 and NP2 as primers and genomic DNA from A02 and AN02 as templates. The expected 1.5-kb *nsdA* fragment was amplified from *S*. *lydicus* A02, while the PCR product from the double-crossover recombination mutant AN02 was 700-bp larger than that from the wild-type A02 (Fig. 5A). These results showed that *nsdA* was replaced precisely with the *tsr* gene in the homologous double-crossover recombination mutant AN02.

The *nsdA*-disrupted mutant AN02 differed from its wild-type strain *S. lydicus* A02 in both pigment production and sporulation. After 10 days of culture on PDA medium, the mutant AN02 produced less yellow pigment than the wild-type A02. Additionally, the mutant AN02 produced only a few light-grey spores, while the wild-type A02 produced abundant dark-grey spores (Fig. 5B).

An HPLC assay of the fermentation products revealed that the disruption of *nsdA* significantly increased the natamycin production of *S*. *lydicus* A02. The mutant AN02 produced nearly 5.0 g L^−1^ natamycin in YSG medium after 96 h, which was increased 1.9-fold compared to the wild-type A02. The changes in natamycin production and physio-morphological features were reversed to the wild-type levels by reintroducing *nsdA* into the mutant AN02 (Fig. 5C). These results showed that *nsdA* repressed the production of natamycin in *S. lydicus* A02.

In our previous study, natamycin production in *S. lydicus* was significantly enhanced (3.0-fold higher) by overexpression of the *slnM* gene in an engineered strain AM02^1^. Here, we found that the transcriptional level of *nsdA* in the wild-type A02 was substantially higher than in AM02 (Supplementary Fig. S6). To test how *nsdA* regulates natamycin production in *S*. *lydicus*, gene expression analysis of the sln cluster in the wild-type A02 and *nsdA* mutant AN02 was performed by qRT-PCR. As the pathway-specific positive regulators of the sln cluster, the transcription of *slnM* in AN02 was much higher than in A02, while the transcript amount of *slnR* was decreased in AN02 (Fig. 5D). This transcription pattern was consistent with the previous finding for *pimM* and *pimR*, the first regulatory genes for pimaricin biosynthesis that function independently of each other^10^. Compared with the wild-type A02, the expression levels of most genes in the sln cluster were increased in AN02, with the exceptions of *slnR*, *slnS0*, *slnS3* and *slnS4*, whose transcript amounts were decreased. Thus, *nsdA* negatively regulates the expression of the sln cluster, directly or indirectly, to repress natamycin biosynthesis in *S*. *lydicus* A02, and the exact mechanism requires further study.

## Conclusions

We presented the first description of the complete genome of natamycin-producing strain A02 by a combination of next-generation sequencing platforms and SMRT sequencing. Comparative genomics analysis based on the core-pan genome, gene families and ANI value showed that *Streptomyces* species that can produce natamycin are associated with genetic recombination more frequently, and they share closer genome evolution compared with other *Streptomyces* species. Comparative analysis of natamycin BGC supported findings from phylogenetic analyses that the natamycin BGCs between *S*. *lydicus* A02 and *S*. *chattanoogensis* L10 showed significantly high similarity, suggesting that the same natamycin biosynthetic regulation mechanisms are used by these two strains. The prediction of secondary metabolite biosynthetic gene clusters and TCS in the genome indicated that strain A02 possesses complex regulatory systems and a high diversity of metabolites. Regulatory analyses of the *phoR-phoP* TCS and *nsdA* gene confirmed phosphate sensitivity and the global negative regulation of natamycin production. The genome sequence and analyses presented in this study provide an important molecular basis for research into natamycin production in *Streptomyces*, which could facilitate rational genome modification to improve the industrial use of A02.

## Materials and Methods

### Manipulation of DNA and RNA from A02

Genomic DNA was isolated from A02 with TIANamp Bacteria DNA Kit (Tiangen, Beijing, China) and dissolved in DNAse-free double-distilled water. For RNA extraction, the wild-type A02 and recombinant strains of *S*. *lydicus* were inoculated into YEME medium without sucrose^3^. Mycelia were collected after 48 h of growth, flash-frozen in liquid nitrogen, and ground into a fine powder. RNA was extracted using the RNeasy Mini Kit (Tiangen) according to the manufacturer’s instructions and treated with DNase I (Tiangen) to eliminate possible chromosomal DNA contamination. The concentration and purity of the DNA and RNA were determined with a NanoDrop 2000 spectrophotometer (Thermo Fisher Scientific, Wilmington, DE, USA).

### Genome sequencing and assembly

The genomic DNA of A02 was sequenced by combining next-generation sequencing platforms (Illumina paired end, 2*90-bp, and 500-bp insert size) and SMRT sequencing (Pacific Biosystems RS) by the Wuhan Institute of Biotechnology (Wuhan, China). PacBio RS (loBPng) reads were cleaned with sub-reads in the SMRT portal, and only clean reads were included in the subsequent analyses. For assembly of the SMRT sequencing reads, the longest reads were first utilized as seeds to recruit all other short reads for the construction of highly accurate preassembled reads through a consensus procedure with HGAP3^36^. Thereafter, the preassembled reads were constructed by aligning all of the reads to each of the seed reads using BLASR^37^. After the preassembly step, the resulting preassembled reads typically had read accuracies above 99%. Celera Assembler^38^ was then used to assemble all of the clean reads to the preassembly, and pilon was applied to generate the best consensus sequence as the final genome sequence result. The method used for correcting Pacbio RSII assembly using the data from the Illumina MiSeq. 2000 was pilon^39^.

### Gene prediction and annotation

Putative protein-coding sequences were predicted using GLIMMER 3.0^40^. CDS annotation was based on the BLASTP (2.2.26) and GO/IPR: iprscan_4.8 programs with NR^41^, Swiss-Prot^42^, TrEMBL^42^, COG^43^, and KEGG^44^ databases, followed by manual inspection. The tRNA and rRNA genes were predicted with tRNAscan-SE^45^ and RNAmmer^46^, respectively. Secondary metabolite gene clusters were predicted by antiSMASH 2.0^47^. The CRISPR finder (http://crispr.u-psud.fr/Server/) was used for identifying CRISPR/Cas systems^48^.

### Core-Pan and synteny analysis

The genes from the A02 genome were used as the gene pool. Then, the genes predicted by Query samples were BLASTed within the gene pool, and the BLAST results were filtered by their length and identity. The BLAST coverage ratios (BCR) of genes from the gene pool and Query samples were calculated separately. If the BCR values from the reference and Query sample were smaller than the setting value, then the gene from the reference was not in homology with the Query, and the gene from the Query genome was added to the gene pool. Query samples had the previous steps repeated one by one, and the final gene pool was called the pan gene pool^49^.

Synteny analysis was performed according to Kurtz *et al*.^50^. The query genome was mapped to the reference genome with MUMmer at the nucleic acid level. The sequence of the target bacterium was ordered according to that of the reference bacterium based on MUMmer. The x and y axes of the two-dimensional synteny graph and the upper and following axes of the linear synteny graph were then constructed after the same proportion of size reduction in the length of both sequences.

### Strains and plasmids

The wild-type A02 and recombinant strains were grown at 29 °C on potato dextrose agar (PDA) slants for spore formation. *S*. *natalensis* ATCC 27448 and *S*. *chattanoogensis* NRRL B-2255 were obtained from Agricultural Research Service Culture Collection. *Escherichia coli* strain DH5α was used as a host for genetic manipulation. Non-methylating *E*. *coli* strain ET12567 (pUZ8002) was used for DNA conjugal transfer from *E. coli* to *S*. *lydicus*. Conjugation and regeneration were performed as described by Kitani *et al*.^51^ and Paranthaman *et al*.^52^. Plasmid pUC19 was used for routine cloning and subcloning experiments. *E*. *coli*-*Streptomyces* shuttle vector pKC1139^53^, containing *oriT* of RK2 and an apramycin resistance gene for selection in actinomycetes and *E. coli*, was used to construct deletion mutants. The integration vector pSET152 containing Φ31 *int* and *attP* ^54^ was used to introduce a single copy of *nsdA* into *S*. *lydicus*.

When necessary, media were supplemented with antibiotics at the following concentrations: 100 μg mL^−1^ apramycin to LB medium, 60 μg mL^−1^ to selection medium, 30 μg mL^−1^ to fermentation medium, and 30 μg mL^−1^ thiostrepton to selection medium. The synthesis of oligonucleotide primers and DNA sequencing of PCR products were performed by Invitrogen (Beijing, China), and all the primers sequences are listed in Supplementary Table S10.

### Construction of a *phoP*-disrupted strain and a *phoP*-complemented strain

To obtain the *phoP-*disrupted mutant, two DNA fragments containing the upstream fragment of 1.045-kb with primers P1 and P2 and downstream fragment of 1.028-kb with primers P3 and P4 were amplified individually. The 1.695-kb *tsr* gene was PCR-amplified from pPM927 with primers TP1 and TP2. These three fragments were sequentially ligated into pKC1139, which was digested with the corresponding enzyme to generate pKC1139P^-^ and verified by PCR and restriction enzyme analysis. The recombinant vector was introduced into *S*. *lydicus* A02 by intergenic conjugation. The *phoP*-deleted mutant AP02 was detected by apramycin sensitivity and thiostrepton resistance tests and confirmed by PCR analysis with the primers PP1 and PP2. GenBank accession numbers for the *phoP* and *phoR* of strain A02 are T2613482 and T2613483, respectively.

### Construction of an *nsdA*-disrupted strain and an *nsdA*-complemented strain

A 1.479-kb coding region of *nsdA* was PCR-amplified from A02 with primers NP1 and NP2, which were designed according to the annotated sequence of *nsdA* from *S*. *lydicus* A02. After being verified by DNA sequencing, the PCR product of *nsdA* was cloned into the vector pMD18-T and then sequenced.

The construction of the *nsdA-*disrupted mutant was performed according to the above method, and the related primer sequences are listed in Supplementary Table S10. To complement the *nsdA* mutant, a 2.2-kb DNA fragment carrying the promoter and coding region of *nsdA* was PCR-amplified with primers NP3 and NP4 using the genomic DNA of A02 as the template. The PCR amplicon was inserted into the *Eco*RI and *Xba*I sites of pSET152 to give pSN152. The resulting vector was introduced into the *nsdA*-disrupted mutant AN02 and further integrated into the chromosome after transformation. The transformants were selected by 60 μg mL^−1^ apramycin and further confirmed by PCR analysis with the primers NP1 and NP2.

### Quantitative analysis of natamycin production by fermentation

Spores (5 × 10^7^ each) of *S*. *lydicus* from PDA agar slant were inoculated into 50 mL of a seed culture medium. The seed cultures were incubated at 29 °C for 24 h on a rotary shaker (250 rpm). Yeast-salts-glycerol (YSG) medium^12^ was used for natamycin production according to Wu *et al*.^1^. Following sample extraction, quantitative analysis of natamycin was carried out using JAI LC-9101 Recycling Preparative HPLC (Japan Analytical Industry, Tokyo, Japan) equipped with a JAIGEL-ODS C18 reverse phase column (Japan Analytical Industry).

### Gene expression analysis by quantitative real-time PCR (qRT-PCR)

qRT-PCR was used to quantify the mRNA levels of natamycin BGC. Primer pairs for qRT-PCR were used according to Lee *et al*.^14^. One microgram of total RNA was used for complementary DNA (cDNA) synthesis, and qRT-PCR analysis was carried out with Takara SYBR^®^ Premix Ex Taq™ II (Tli RNaseH Plus) (Takara Beijing, China) supplemented with ROX using an ABI GeneAmp PCR System 7500. The *lysA* gene was used as the reference gene. The primers for the *slnL* gene were designed using Primer 5 software (PREMIER Biosoft International, Silicon Valley, USA) (Table S10).

### Data availability

The chromosome sequence of A02 using next-generation sequencing platforms and SMRT sequencing was deposited in Genbank under the accession number CP007699.1 and CP007699.2.

## Electronic supplementary material

Supplementary information
