# Supplementary material for: Comparative Genomic and Regulatory Analyses of Natamycin Production of Streptomyces lydicus A02
Source: Sci Rep. 2017 Aug 22;7:9114. doi: 10.1038/s41598-017-09532-3 (PMC5567329; doi:10.1038/s41598-017-09532-3)
Supplement: Supplementary file 1 — Supplementary information [file 41598_2017_9532_MOESM1_ESM.doc]

**Supplementary Figures and Tables**

**Comparative Genomic and Regulatory Analyses of Natamycin Production of *Streptomyces lydicus* A02**

**Huiling Wu^1^ Weicheng Liu^1^* Lingling Shi^1^ Kaiwei Si^3^ Ting Liu^1^ Dan Dong^1^  Taotao Zhang^1^ Juan Zhao^1^ Dewen Liu^1^ Zhaofeng Tian^1^ Yuesen Yue^2^* Hong Zhang^1^ Bai Xuelian^1^ Yong Liang^3^**

^1^ Institute of Plant and Environment Protection, Beijing Academy of Agriculture and Forestry Sciences, Beijing 100097, China

^2^ Beijing Research and Development Center for Grass and Environment, Beijing Academy of Agriculture and Forestry Sciences, Beijing 100097, China

^3^ BGI-Shenzhen, Shenzhen, Guangdong, 518083, China

**Title running head: Genome sequencing and natamycin biosynthetic regulation**

*** Corresponding author**

E-mail: liuwich@163.com (WCL)

yysen2008@sina.com (YSY)

Supplementary Figures

**Figure S1** Morphology, pigments (A) and natamycin production (B), and gene expression of natamycin cluster (C) in wild type A02, *S*. *natalensis* ATCC 27448 and *S*. *chattanoogensis* NRRL B-2255.

A

B


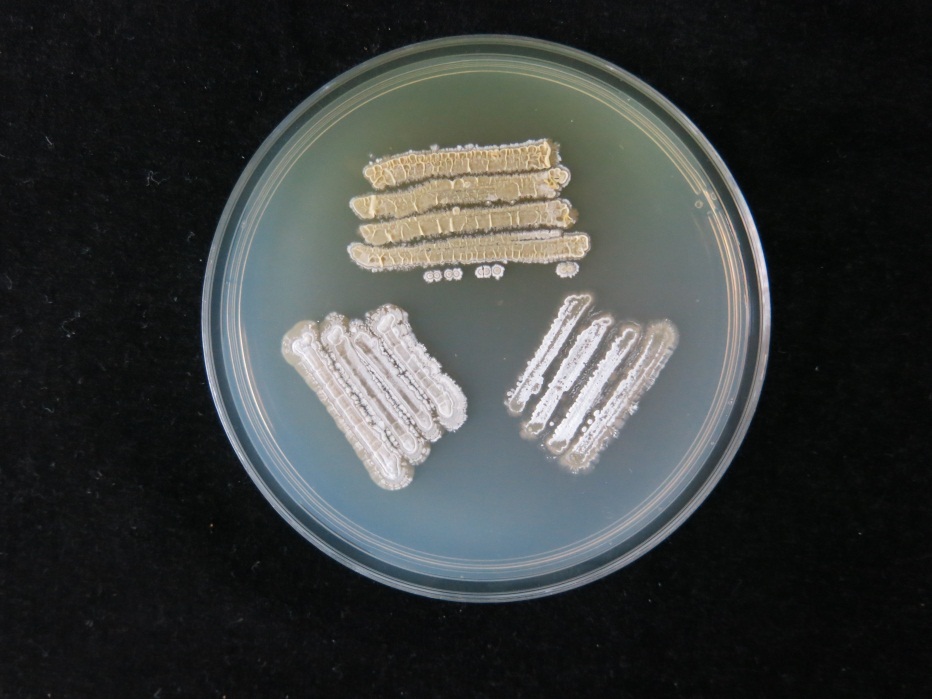

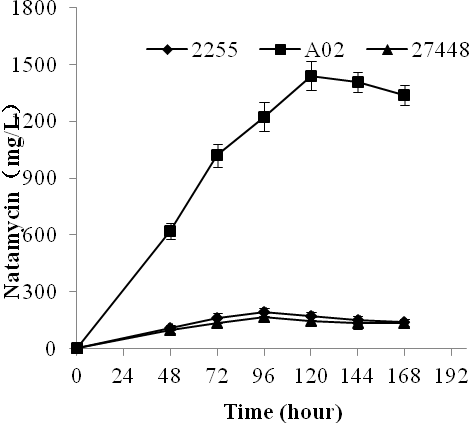


**2255**

**A02 27448**

C


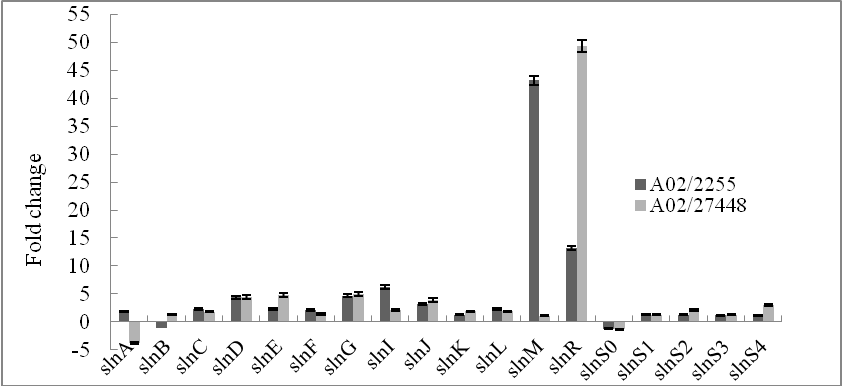


**Figure S2** Gene length distribution of *S*. *lydicus* A02.

Gene length (bp)


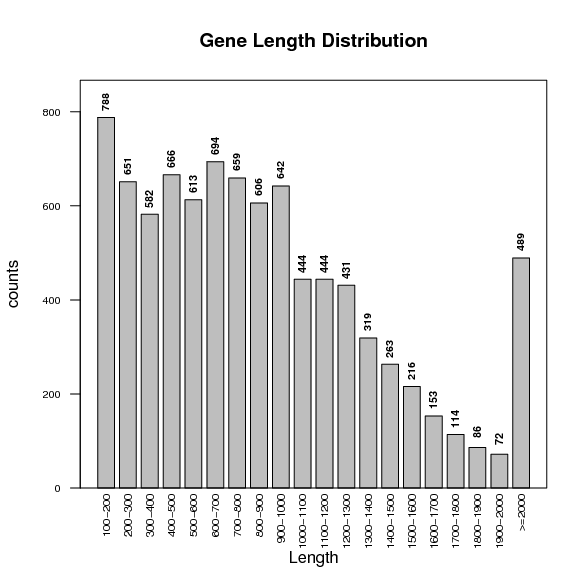


**Figure S3** Schematic representation of the *S*. *lydicus* A02 chromosome. From outer to inner, circle the 1st and 2nd (negative and positive strands) shows the COG function of negative strand along the chromosome that each color represents a function classification; the 3rd circle shows ncRNA: sRNA (grey), tRNA (red), rRNA (purple); the 4rd circle shows crispr array (blue) and cas (crispr-association) protein (red); the 5th circle shows the GC content (black); the 6th circle shows the GC skew ( (G-C)/(G+C) , green > 0, purple < 0). The 5th circle and the 6th circle are plotted relative to the average value.


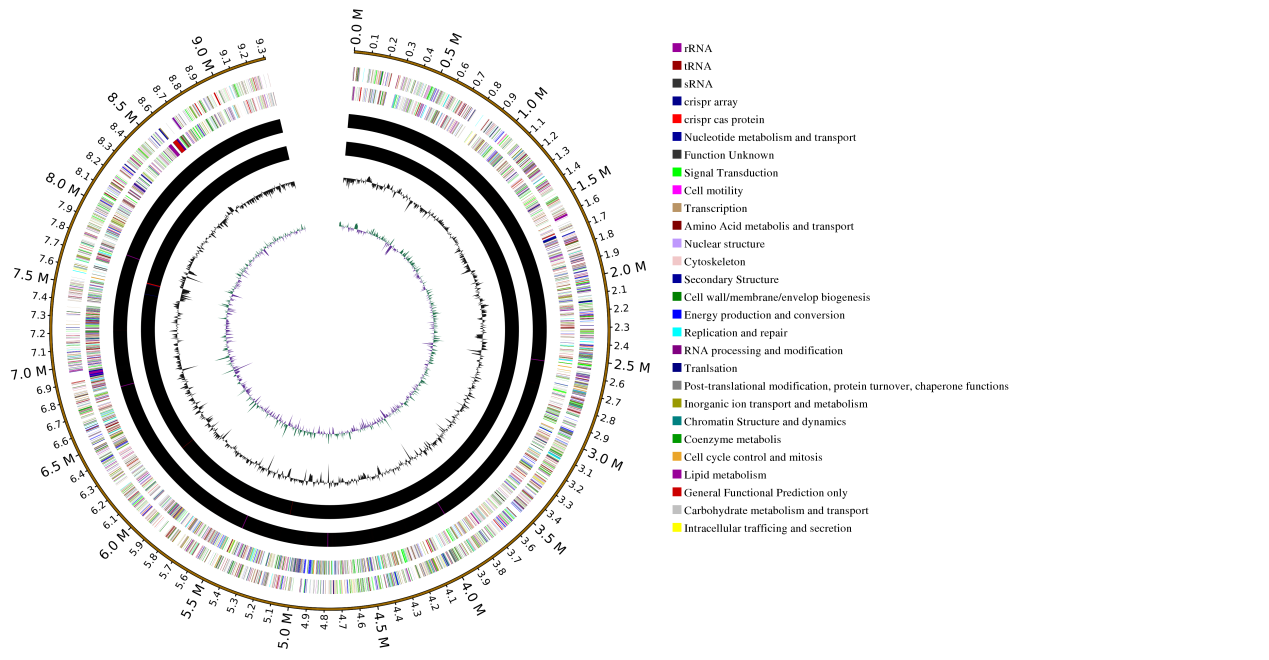


**Figure S4** The second CRISPR locus of *S*. *lydicus*A02. The CRISPR element is presented in pink and the CRISPR associated genes (Cas) are presented in light blue. Within CRISPR, the diamond represents complete repeat, the triangle represents incomplete repeat, and the square indicates spacer.

**Integrase**

**Lactoylglutathionelyase**

**CRISPR**

**Transcriptionalregulator**

1 Kb

**Cas4**

**Cas5**

**0.7 0.4**

**Cas1**

**Cas2**

**Cas3**

**Cas3**

**1 1.7 0.6 0.7 0.8 3**

**1.2**

**Complete Repeat**

**Incomplete Repeat**

**Spacer**

**Complete Repeat sequence**

**GGGAACACCCCCGCAGGCGCGGGGACCAC**

**Specific spacer sequences**

**Spacer 12**

**GGAACCACGCAGGCGCGGGGACCACCGACTCGCGGCCGGCGTACTGCGTCCGGGCCA**

**Spacer 19**

**CCAGGGCAGCACCGTCACAGGGAT**

**Spacer 20**

**CACCCCCGCAGTCGCGGGGACCACCCGGTACTCCGTGTAGGACAGGCCGGTGGTCA**

**Figure S5** Heatmap after core gene deletion.

*
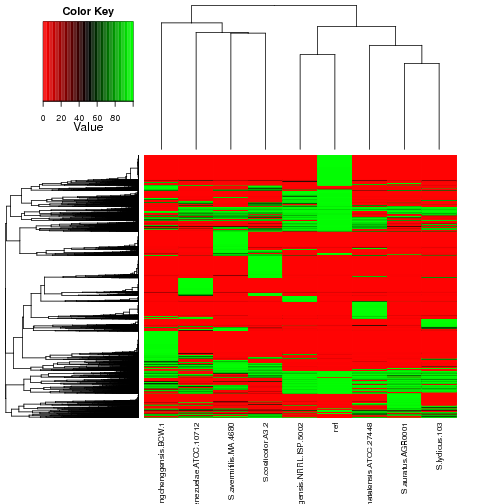
*

*S*. *bingchenggensis* BCW-1

*S*. *venezuelae* ATCC 10712

*S*. *avermitilis* MA-4680

*S*. *coelicolor*A3(2)

*S*. *chattanoogensis* NRRL ISP5002

A02

*S*. *natalensis* ATCC 27448

*S*. *auratus* AGR0001

*S*. *lydicus* 103

*S*.*lydicus*103

**Figure S6** The transcriptional levels of *nsdA* in strains A02 and AM02.The *hrdB* gene was used as internal control.


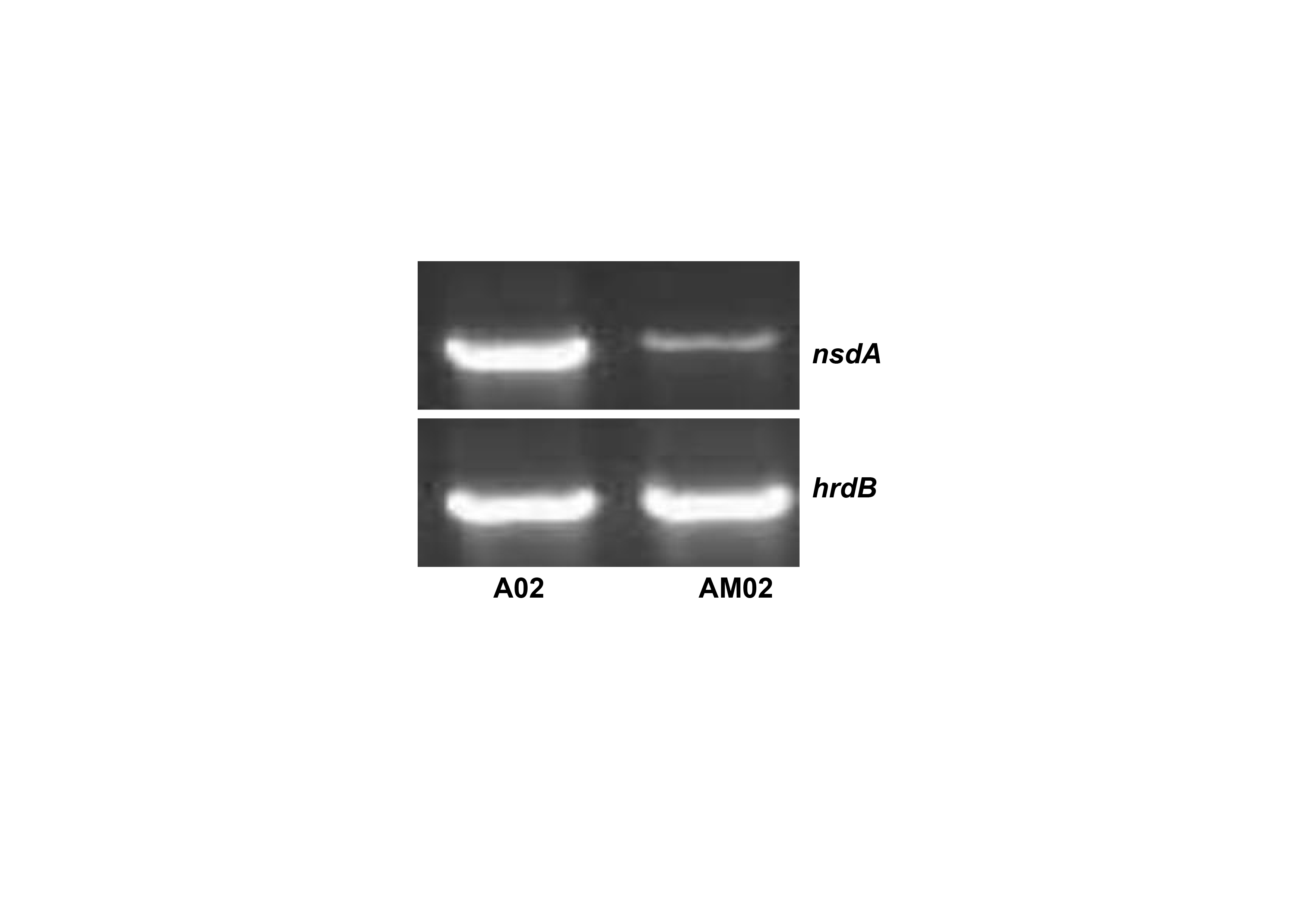


**Table S1** Characteristics of the *S*. *lydicus*A02 genome assembly in Hiseq 2000 sequencing platform.

| **Content** | **Scaffold** | | **Contigs** | | |
| --- | --- | --- | --- | --- | --- |
| Total number | | 1 | | 1 |  |
| Total length (bp) | | 9,300,345 | | 9,300,318 | |
| Gap length (bp) | | 27 | | 0 | |
| Average length (bp) | | 9,300,345 | | 2325.079,50 | |
| N50 (bp) | | 9,300,345 | | 5,402,844 | |
| N90 (bp) | | 9,300,345 | | 840,722 | |
| Max length (bp) | | 9,300,345 | | 5,402,844 | |
| Min length (bp) | | 9,300,345 | | 409,783 | |
| Sequence GC (%) | | 70.70 | | 70.70 | |

**Table S2** The sequencing data of genome in *S*. *lydicus* A02. The genome of *S*. *lydicus* A02 was sequenced and produced 593.1 Mb data by using SMRT sequencing.

| Sample Name | Polymerase Reads Number | Polymerase Reads Mean Length(bp) | Polymerase Reads Bases(bp) | Polymerase Reads Quality | Subreads Number | Subreads Mean Length(bp) | Subreads Bases(bp) | Subreads Quality | Utilization Ratio |
| --- | --- | --- | --- | --- | --- | --- | --- | --- | --- |
| A02 | 43,361 | 13,607 | 1,650,686,412 | 0.86 | 80,754 | 7,345 | 593,142,840 | 0.86 | 0.39 |

**Table S3** Number of genes associated with the general COG functional categories.

| **Code** | **Value** | **Percentage** | **Description** |
| --- | --- | --- | --- |
| A | 2 | 0.0 | RNA processing and modification |
| B | 2 | 0.0 | Chromatin structure and dynamics |
| C | 345 | 3.9 | Energy production and conversion |
| D | 77 | 0.9 | Cell cycle control, cell division, chromosome partitioning |
| E | 580 | 6.5 | Amino acid transport and metabolism |
| F | 152 | 1.7 | Nucleotide transport and metabolism |
| G | 475 | 5.3 | Carbohydrate transport and metabolism |
| H | 359 | 4.0 | Coenzyme transport and metabolism |
| I | 412 | 4.6 | Lipid transport and metabolism |
| J | 306 | 3.4 | Translation, ribosomal structure and biogenesis |
| K | 839 | 9.4 | Transcription |
| L | 177 | 2.0 | Replication, recombination and repair |
| M | 306 | 3.4 | Cell wall/membrane/envelope biogenesis |
| N | 21 | 0.2 | Cell motility |
| O | 221 | 2.5 | Posttranslational modification, protein turnover, chaperones |
| P | 317 | 3.5 | Inorganic ion transport and metabolism |
| Q | 315 | 3.6 | Secondary metabolites biosynthesis, transport and catabolism |
| R | 825 | 9.3 | General function prediction only |
| S | 291 | 3.3 | Function unknown |
| T | 501 | 5.6 | Signal transduction mechanisms |
| U | 58 | 0.7 | Intracellular trafficking, secretion, and vesicular transport |
| V  W  X | 204  11  127 | 2.3  0  1.4 | Defense mechanisms  Extracellular structures  Mobilome: prophages, transposons |
| -- | 1965 | 22 | Not in COGs |

**Table S4** The ANI value between A02 and different *Streptomyces* species.

| Species | ANI value (%) |
| --- | --- |
| *S*. *auratus* AGR0001 | 85.2385 |
| *S*.*avermitilis MA-4680* | 77.7476 |
| *S*.*bingchenggensis* BCW-1 | 78.4623 |
| *S*.*chattanoogensis*NRRL ISP-5002 | 97.4181 |
| *S*.*coelicolor* A3(2) | 77.4893 |
| *S*.*natalensis* ATCC 27448 | 87.8496 |
| *S.venezuelae* ATCC 10712 | 77.923 |
| *S*.*lydicus* 103 | 84.9077 |

**Table S5 Comparison of the natamycin BGCs from *S*. *lydicus* A02, *S. chattanoogensis* L10 and *S*. *natalensis* ATCC 27448 in amino acid levels.**

| Function | A02 | L10 | 27448 | Similarity withA02 | |
| --- | --- | --- | --- | --- | --- |
|  |  |  |  | L10 | 27448 |
| PKS (loading module) | SlnS0 (1757) | ScnS0 (1755) | PimS0 (1847) | 99% | 93% |
| PKS (modules 1-4) | SlnS1 (6859) | ScnS1 (6831) | PimS1 (6797) | 99% | 93% |
| PKS (modules 5-10) | SlnS2 (9509) | ScnS2 (9498) | ﻿PimS2 (9507) | 99% | 94% |
| PKS (modules 11) | SlnS3 (1806) | ScnS3 (1813) | PimS2 (1808) | 99% | 92% |
| PKS (module 12) | SlnS4 (2021) | ScnS4 (2021) | PimS4 (2024) | 99% | 92% |
| ABC transporter | SlnA (602) | ScnA (602) | PimA (602) | 99% | 93% |
| ABC transporter | SlnB (627) | ScnB (627) | PimB (626) | 99% | 95% |
| Aminotransferase | SlnC (352) | ScnC (352) | PimC (352) | 100% | 97% |
| P450 monooxygenase | SlnD (397) | ScnD (397) | PimD (397) | 99% | 96% |
| P450 monooxygenase | SlnG (395) | ScnG (395) | PimG (398) | 99% | 97% |
| Efflux pump |  |  | PimH (432) |  |  |
| Cholesterol oxidase | SlnE (552) | ScnE (552) | PimE (549) | 100% | 97% |
| Ferredoxin | SlnF (63) | ScnF (63) | PimF (63) | 99% | 98% |
| Thioesterase | SlnI (255) | ScnI (255) | PimI (255) | 99% | 94% |
| Mycosaminedehydratase | SlnJ (343) | ScnJ (285) | PimJ (343) | 100% | 97% |
| Glucosyltransferase | SlnK (458) | ScnK (458) | PimK (458) | 99% | 96% |
| Regulator | SlnRII (192) | ScnRII (192) | PimM (192) | 100% | 96% |
| Regulator | SlnRI (1176) | ScnRI (1176) | PimR (1198) | 99% | 92% |
| Tnp | Tnp (406) | Tnp (406) |  | 97% |  |
| Amino acid exporter |  |  | PimT (214) |  |  |
| Tyrosine phosphatase | SlnL (223) | ScnL (223) |  | 99% |  |

**Table S6 Predicted gene clusters involving in the biosynthesis of secondary metabolites.**

| No. | Product  Type | Protein ID  Start End | | Length  (bp) | | Putative function |
| --- | --- | --- | --- | --- | --- | --- |
| 1 | T2pks | 5 | 28 | 43,505 | Uncultured bacterium arixanthomycin gene cluster (24% of genes show similarity) | |
| 2 | Melanin | 123 | 129 | 10,564 | 38% of genes show similarity with *S*. *lydicus* 103 genome sequence | |
| 3 | Transatpks | 244 | 265 | 82,633 | *S*. *platensis* subsp. *rosaceus* strain NRRL 18993dorrigocin/mi-grastatin gene cluster (75% of genes show similarity) | |
| 4 | Terpene | 371 | 381 | 21311 | 42% of genes show similarity with *S*. *griseochromogenes* ATCC 14511 genome sequence | |
| 5 | [Butyrolactone](http://antismash.secondarymetabolites.org/help#butyrolactone) | 520 | 526 | 10981 | *S*. *chattanoogensis* L10 hypothetical protein (gbdA), butyrolactone receptor (scgR), ScgA (scgA), and ScgX (scgX) (68% of genes show similarity) | |
| 6 | [Ladderane](http://antismash.secondarymetabolites.org/help#ladderane) | 730 | 752 | 41158 | *S*. *chattanoogensis* L10AzoK (azoK) gene (9% of genes show similarity) | |
| 7 | [Other](http://antismash.secondarymetabolites.org/help#other) | 1005 | 1026 | 40714 | 46% of genes show similarity with *S*. *lydicus* 103 genome sequence | |
| 8 | [Terpene](http://antismash.secondarymetabolites.org/help#terpene) | 1052 | 1063 | 21335 | 27% of genes show similarity with *S*. *bingchenggensis* BCW-1 genome sequence | |
| 9 | [T2pks](http://antismash.secondarymetabolites.org/help#t2pks) | 1242 | 1265 | 43520 | 70% of genes show similarity with *S*. *albulus* ZPM genome sequence | |
| 10 | [Terpene](http://antismash.secondarymetabolites.org/help#terpene) | 1265 | 1278 | 30321 | 94% of genes show similarity with *S*. *fulvissimus* DSM 40593 genome sequence | |
| 11 | [Lantipeptide](http://antismash.secondarymetabolites.org/help#lantipeptide) | 1365 | 1379 | 27080 | 21% of genes show similarity with *S*. *noursei* ATCC 11455 genome sequence | |
| 12 | [Terpene](http://antismash.secondarymetabolites.org/help#terpene) | 1458 | 1469 | 21065 | 42% of genes show similarity with *S*. *noursei* ATCC 11455 genome sequence | |
| 13 | [Other](http://antismash.secondarymetabolites.org/help#other) | 1468 | 1490 | 43966 | 7% of genes show similarity with *S*. *noursei* ATCC 11455 genome sequence | |
| 14 | Nrps-T1pks | 1613 | 1639 | 58497 | 16% of genes show similarity with *S*. *lydicus* 103 genome sequence | |
| 15 | [Cyanobactin](http://antismash.secondarymetabolites.org/help#cyanobactin) | 1650 | 1663 | 21113 | 3% of genes show similarity with *S*. *albulus*J1074 genome sequence | |
| 16 | [Nrps](http://antismash.secondarymetabolites.org/help#nrps) | 1703 | 1734 | 68912 | 15% of genes show similarity with *S*. *iranensis* genome sequence | |
| 17 | [T3pks](http://antismash.secondarymetabolites.org/help#t3pks) | 1745 | 1766 | 41062 | 71% of genes show similarity with *S*. *lydicus* 103 genome sequence | |
| 18 | [Lantipeptide](http://antismash.secondarymetabolites.org/help#lantipeptide) | 1795 | 1808 | 26791 | *Planomonospora alba* planosporicin biosynthesis gene cluster, strain NRRL 18924 (83% of genes show similarity) | |
| 19 | [Nrps](http://antismash.secondarymetabolites.org/help#nrps) | 2099 | 2120 | 50792 | 64% of genes show similarity with *Streptomyces* sp.769 genome sequence | |
| 20 | [Bacteriocin](http://antismash.secondarymetabolites.org/help#bacteriocin) | 2128 | 2134 | 10870 | 60% of genes show similarity with *S*. *lydicus* 103 genome sequence | |
| 21 | [Butyrolactone](http://antismash.secondarymetabolites.org/help#butyrolactone) | 2254 | 2259 | 10945 | *S*. *chattanoogensis* L10gamma butyrolacone biosynthesis (69% of genes show similarity) | |
| 22 | [Siderophore](http://antismash.secondarymetabolites.org/help#siderophore) | 2408 | 2415 | 17278 | 96% of genes show similarity with *S*. *noursei* ATCC 11455 genome sequence | |
| 23 | [Terpene](http://antismash.secondarymetabolites.org/help#terpene) | 4014 | 4024 | 22235 | 60% of genes show similarity with *S*. *lydicus* 103 genome sequence | |
| 24 | [Terpene](http://antismash.secondarymetabolites.org/help#terpene) | 5557 | 5567 | 21107 | 74% of genes show similarity with *S*. *lydicus* 103 genome sequence | |
| 25 | [Lassopeptide](http://antismash.secondarymetabolites.org/help#lassopeptide) | 5879 | 5892 | 24391 | 34% of genes show similarity with *S*. *lydicus* 103 genome sequence | |
| 26 | [Lassopeptide](http://antismash.secondarymetabolites.org/help#lassopeptide) | 5991 | 6000 | 14769 | 6% of genes show similarity with *Streptomyces* sp. CNQ-509 genome sequence | |
| 27 | Ectoine | 6444 | 6450 | 10414 | Ectoine biosynthetic gene cluster (96% of genes show similarity) | |
| 28 | [Siderophore](http://antismash.secondarymetabolites.org/help#siderophore) | 6533 | 6538 | 11797 | 92% of genes show similarity with *S*. *lydicus* 103 genome sequence | |
| 29 | [T1pks](http://antismash.secondarymetabolites.org/help#t1pks) | 6625 | 6651 | 84828 | 47% of genes show similarity with *Streptomyces* sp. 769 genome sequence | |
| 30 | [T2pks](http://antismash.secondarymetabolites.org/help#t2pks) | 6993 | 7015 | 43490 | *S*. *chattanoogensis* L10 chattamycin biosynthetic gene cluster (78% of genes show similarity) | |
| 31 | [Oligosaccharide](http://antismash.secondarymetabolites.org/help#oligosaccharide) | 7016 | 7040 | 48836 | *S. chattanoogensis* L10 chattamycin biosynthetic gene cluster (77% of genes show similarity) | |
| 32 | [Siderophore](http://antismash.secondarymetabolites.org/help#siderophore) | 7319 | 7325 | 17112 | 68% of genes show similarity with *S*. *avermitilis* MA-4680 genome sequence | |
| 33 | Lantipeptide-Bacteriocin | 7554 | 7568 | 34404 | 55% of genes show similarity with *S*. *lydicus* 103 genome sequence | |
| 34 | Nrps-Lassopeptide | 7816 | 7850 | 69234 | 59% of genes show similarity with *Streptomyces* sp. CNQ-509 genome sequence | |
| 35 | [Nrps](http://antismash.secondarymetabolites.org/help#nrps) | 7871 | 7901 | 66339 | 21% of genes show similarity with *S*. *lydicus* 103 genome sequence | |
| 36 | [T1pks](http://antismash.secondarymetabolites.org/help#t1pks) | 8151 | 8170 | 86194 | *S*. *chattanoogensis* L10 natamycin biosynthetic gene cluster (99% of genes show similarity) | |
| 37 | [T1pks](http://antismash.secondarymetabolites.org/help#t1pks) | 8196 | 8217 | 44305 | 33% of genes show similarity with *S*. *violaceusniger* Tu 4113 genome sequence | |
| 38 | [Lantipeptide](http://antismash.secondarymetabolites.org/help#lantipeptide) | 8322 | 8333 | 22745 | 59% of genes show similarity with *S*. *lydicus* 103 genome sequence | |
| 39 | [Other](http://antismash.secondarymetabolites.org/help#other) | 8701 | 8721 | 41371 | 77% of genes show similarity with *S*. *lydicus* 103 genome sequence | |
| 40 | Lantipeptide-Lassopeptide | 8719 | 8742 | 42706 | 15% of genes show similarity with *S*. *collinus* Tu 3651 genome sequence | |

**Table S7** Two-component signal transduction systems in *S*. *lydicus* A02.

| TCS gene (hk/rr) | Subfamily |
| --- | --- |
| A02_0577/0579, A02_1303/1304, A02_1367/1369, A02_1499/1500,A02_2117/2119, A02_2377/2378, A02_2548/2550, A02_2756/2757, A02_2821/2822, A02_3063/3065, A02_3452/3453, A02_3582/3583, A02_3831/3832, A02_3977/3978, A02_4253/4254, A02_4440/4441, A02_4460/4461,A02_4505/4506, A02_4549/4551, A02_4582/4583, A02_5830/5831, A02_5930/5932, A02_6166/6167, A02_6206/6207, A02_6439/6440, A02_6519/6521, A02_7106/7107, A02_8138/8139,A02_8294/8295, A02_8389/8390, and A02_8771/8772 | NarL |
| A02_0272/0274, A02_1342/1344, A02_1381/1383, A02_1489/1490, A02_1519/1520, A02_2266/2268, A02_2279/2280, A02_2434/2435, A02_2738/2740, A02_2783/2785, A02_2796/2798, A02_2878/2879, A02_3267/3268, A02_3630/3632, A02_3686/3687, A02_3803/3804, A02_4091/4092, A02_4399/4440, A02_5178/5179, A02_5231/5232, A02_5439/5440, A02_5849/5851, A02_6714/6716, A02_7545/7547, A02_8642/8643, and A02_8747/8748 | OmpR |
| A02_2582/2583 | AmtB |
| A02_2372/2374, A02_2764/2766, and A02_7621/7623 | CitB |

**Table S8** Comparison of *phoP*/*phoR* homologous genes in different *Streptomyces* species.

| Species | GenBank numbers | Length (bp) | Similarity with *phoP*/*phoR* of A02 | |
| --- | --- | --- | --- | --- |
|  |  |  | Amino acid (%) | Nucleotide (%) |
| *S*. *lydicus*A02 | T2613482/3 | 672/1272 | 100/100 | 100/100 |
| *S*. *natalensis* | AM176576.1 | 672/1284 | 99/97 | 96/94 |
| *S*. *avermitilis* MA-4680 | SAV3972/3 | 672/1278 | 98/88 | 90/85 |
| *S*. *lividans* | AJ544582.1 | 672/1281 | 97/86 | 90/86 |
| *S*. *coelicolor* A3(2) | SCO4229/30 | 672/1281 | 97/87 | 90/86 |
| *S*. *albulus* NK660 | DC744736/7 | 681/1269 | 98/96 | 94/92 |
| *S*. *hygroscopicus* 5008 | SHJG4863/4 | 681/1296 | 97/85 | 91/88 |
| *S*. *griseus* | SGR4008/9 | 681/1263 | 98/83 | 91/85 |
| *S*. *bingchenggensis* BCW-1 | SBI04993/4 | 681/1290 | 97/88 | 89/88 |

**Table S9** Comparison of *nsdA* homologous genes in different *Streptomyces* species.

| Species | GenBank numbers | Length of gene (bp) | Similarity with *nsdA* of *S*. *lydicus*A02 | |
| --- | --- | --- | --- | --- |
|  |  |  | Amino acid (%) | Nucleotide (%) |
| *Streptomyceslydicus*A02 | CP007699.2 | 1479 | 100 | 100 |
| *S*. *qingfengmyceticus* | DQ478680.1 | 1503 | 88 | 91 |
| *S*. *bingchenggensis* BCW-1 | SBI_03475 | 1500 | 84 | 80 |
| *S*. *lividans* | DQ478681.1 | 1503 | 81 | 75 |
| *S*. *avermitilis* MA-4680 | SAV2652 | 1476 | 82 | 81 |
| *S*. *hygroscopicus* 5008 | DQ478679.1 | 1479 | 82 | 79 |
| *S*. *coelicolor* A3(2) | SCO5582 | 1503 | 81 | 75 |
| *S*. *albulus* | CP007574.1 | 1473 | 83 | 80 |
| *S*. *bingchengensis* 226541 | EU779992.2 | 1485 | 80 | 76 |

**Table S10** Primers used in this study.

| Primer | Sequence (5'–3') | Description |  |
| --- | --- | --- | --- |
| P1 | (*Eco*RI) GGAATTCTGATGGACGCCAGCGACG | Forward primer for upstream of *phoP* | |
| P2 | (*Eco*RV) TGCAGGATATCGCGGGAGGAGAAGGGCTTG | Reverse primer for upstream of *phoP* | |
| P3 | (*Xba*I) GCTCTAGATCTGCCGCTCAAGGAGTT | Forward primer for downstream of *phoP* | |
| P4 | (*Hin*dIII) CCCAAGCTTGCTCGTCAAGGAGATGCC | Reverse primer for downstream of *phoP* | |
| TP1 | (*Xba*I) GCTCTAGAGGTACCCGATC | Forward primer for thiostrepton resistance gene (*tsr*) | |
| TP2 | (*Xba*I) GCTCTAGACGCCGACCGGGACCCGCA | Reverse primer for thiostrepton resistance gene (*tsr*) | |
| PP1 | CGTGACCCGAGT GCTTGTC | Forward primer for *phoP* | |
| PP2 | TTACGGCTCGAACTTGTAC | Reverse primer for *phoP* | |
| NP1 | GTGGCCGGCAACGGCGGC | Forward primer for *nsdA* | |
| NP2 | TCAGACGGCCTCCATCGCG | Reverse primer for *nsdA* | |
| UP1 | (*Eco*RI) GGAATTCGCTCCTTGCCGAAGTCCA | Forward primer for upstream of *nsdA* | |
| UP2 | (*Eco*RV) TGCAGGATATCTGAGGTTGACCGTCTGGG | Reverse primer for upstream of *nsdA* | |
| DP1 | (*Xba*I) GCTCTAGACGTTCGCCAAACAGGTCC | Forward primer for downstream of *nsdA* | |
| DP2 | (*Hin*dIII) CCCAAGCTTCGCATCGGGTCCTTCTTG | Reverse primer for downstream of *nsdA* | |
| NP3 | (*Eco*RI) GGAATTCGGGCGACGAAGAACAGCG | Forward primer for *nsdA* with native promoter | |
| NP4 | (*Xba*I) GCTCTAGATCAGACGGCCTCCATCGCG | Reverse primer for *nsdA* with native promoter | |
| NP5 | TCCAAGCGGCTGTTCAAG | RT-PCR forward primer for *nsdA* | |
| NP6 | CCTCGGAGAAGAAGCGGAT | RT-PCR reverse primer for *nsdA* | |
| LP1 | GGCTCGTTCACCGTGTCCT | Forward primer of *slnL*for qRT-PCR | |
| LP2 | ACGCCCATCCTCACCCA | Reverse primer of *slnL*for qRT-PCR | |
